# Perturbed rhythmic activation of signaling pathways in mice deficient for Sterol Carrier Protein 2-dependent diurnal lipid transport and metabolism

**DOI:** 10.1038/srep24631

**Published:** 2016-04-21

**Authors:** Céline Jouffe, Cédric Gobet, Eva Martin, Sylviane Métairon, Delphine Morin-Rivron, Mojgan Masoodi, Frédéric Gachon

**Affiliations:** 1 Department of Pharmacology and Toxicology, University of Lausanne, Lausanne, CH-1011, Switzerland; 2 Department of Diabetes and Circadian Rhythms, Nestlé Institute of Health Sciences, CH-1015 Lausanne, Switzerland; 3 Faculty of Life Sciences, Ecole Polytechnique Fédérale de Lausanne (EPFL), CH-1015 Lausanne, Switzerland; 4 Functional Genomic, Nestlé Institute of Health Sciences, CH-1015 Lausanne, Switzerland; 5 Department of Gastro-Intestinal Health & Microbiome, Nestlé Institute of Health Sciences, CH-1015 Lausanne, Switzerland; 6 Department of Nutritional Sciences, Faculty of Medicine, University of Toronto, Toronto, ON, M5S 3E2, Canada

## Abstract

Through evolution, most of the living species have acquired a time keeping system to anticipate daily changes caused by the rotation of the Earth. In all of the systems this pacemaker is based on a molecular transcriptional/translational negative feedback loop able to generate rhythmic gene expression with a period close to 24 hours. Recent evidences suggest that post-transcriptional regulations activated mostly by systemic cues play a fundamental role in the process, fine tuning the time keeping system and linking it to animal physiology. Among these signals, we consider the role of lipid transport and metabolism regulated by SCP2. Mice harboring a deletion of the *Scp2 *locus present a modulated diurnal accumulation of lipids in the liver and a perturbed activation of several signaling pathways including PPARα, SREBP, LRH-1, TORC1 and its upstream regulators. This defect in signaling pathways activation feedbacks upon the clock by lengthening the circadian period of animals through post-translational regulation of core clock regulators, showing that rhythmic lipid transport is a major player in the establishment of rhythmic mRNA and protein expression landscape.

As a result of living in an environment subjected to light-dark cycles caused by Earth’s rotation, organisms from bacteria to mammals have acquired a timing system allowing anticipation of these diurnal variations. In mammals, this timer is called the circadian clock (*circadian* meaning about a day) and influences most aspects of physiology and behavior^1^. As a consequence, perturbations or misalignments of the circadian clock in humans, for example in the case of shift-workers, lead to diverse pathologies including metabolic disorders and obesity^2^. If the oscillatory timing system is cell-autonomous, timing at the organism scale is based on a hierarchical organization. Indeed, a “master clock” within the Suprachiasmatic Nucleus (SCN) of the hypothalamus, which is resynchronized daily through light input, communicates timing signals to “slave” oscillators in other peripheral tissues which are more sensitive to systemic signals like metabolic cues coming from food^3^.

In mammals, the molecular oscillator consists of interconnected transcriptional and translational feedback loops. Briefly, the transcriptional activator consists in the transcription factor protein Brain and Muscle Aryl hydrocarbon receptor nuclear translocator-Like 1 (BMAL1) which can form heterodimers with the proteins Circadian Locomotor Output Cycles Kaput (CLOCK) or Neuronal PAS domain-containing protein 2 (NPAS2). This heterodimer activates, *via* E-box elements, the transcription of the negative feedback loop components, namely PERIOD (PER1-2) and CRYPTOCHROME (CRY1-2). PERs and CRYs dimerize and the resulting complex represses the transcriptional activity of the BMAL1 complex. The BMAL1 complex also activates the expression of an additional loop involving REV-ERBs and Retinoic acid receptor-related Orphan Receptors (RORs) proteins. REV-ERBs and RORs compete for the access of ROR elements located within the *Bmal1* gene promoter. They repress and activate *Bmal1* expression, respectively, thereby reinforcing the stability and the precision of the circadian oscillator (for a review see^4^). It is now evident that additional layers of control including temporal post-transcriptional and post-translational regulations play a critical role in the establishment of a stable clock. This additional layer of regulation is largely regulated by systemic signals coming from circadian clock and/or feeding coordinated rhythmic metabolism, allowing in this way the adjustment of the molecular clockwork with the metabolic state of the cell^5^. At least in part, this effect is mediated through the rhythmic activation of signaling pathways that modulates the activity of core clock regulators. In parallel these rhythmically activated signaling pathways can also feed back on metabolism. We have for example recently shown that circadian-clock orchestrated liver metabolism regulates rhythmic activation of the Unfolded Protein Response (UPR)^6^ and ribosome biogenesis through rhythmic activation of mRNA translation via the Target of Rapamycin Complex 1 (TORC1) pathway and its upstream regulators PhosphatidylInositol-4,5-bisphosphate 3-Kinase (PI3K), Extracellular-signal-Regulated Kinases (ERK), and 5′ Adenosine Monophosphate-activated Protein Kinase (AMPK)^7^.

To gain more insight into the metabolic pathways involved in this process, we speculate about the potential role of rhythmic lipid transport. Indeed, recent evidences showed that lipid metabolism and transport are key modulators of the activation of numerous signaling pathways including the UPR^8^, TORC1^9^, or ERK^10^. This modulation of activation often involves lipid-dependent organization of membrane proteins in lipid rafts^11^ which produces changes in their structure and potential activation^12^. In parallel, several signaling pathways such as PI3K^13^, Peroxisome Proliferator-Activated Receptor α (PPARα)^14^, Liver Receptor Homolog-1 (LRH-1)^15^, Liver X Receptor (LXR)^16^, and Sterol Regulatory Element-Binding Protein (SREBP)^17^ are directly regulated by lipid metabolism. Interestingly, PPARα and SREBP exhibit a rhythmic activation caused by interconnection between circadian clock and rhythmic lipid metabolism^18^,^19^.

The aim of our study was to investigate the potential influence of rhythmic liver lipid transport on the activation of these pathways. Sterol Carrier Protein 2 (SCP2) is an intracellular lipid transporter that presents a diurnal expression at the protein level in mouse liver^20^. This rhythmic expression is regulated at the post-transcriptional level as *Scp2* mRNA does not present rhythmic expression^21^. SCP2 is involved in the transfer of several lipid species from the endoplasmic reticulum (ER) where they are synthesized to the plasma membrane^22^, affecting in this way the formation of lipid rafts and cell signaling (for a review see^23^). The *Scp2* locus also encodes for the *Scpx* mRNA through alternative transcription start site usage, the later encoded for protein presenting peroxisomal 3-ketoacyl-CoA thiolase activity involved in the oxidation of branched-chain lipids^24^. As a consequence, lipid metabolism^25^ and expression of proteins at the plasma membrane in lipid raft domains are perturbed in *Scp2* deficient animals^26^. Although the differential role of the two proteins encoded by the *Scp2* locus in the process is not clearly established yet, *in vitro* experiments showed that the lipid transport activity of SCP2 is required^27^.

We speculate that SCP2 could affect diurnal liver lipid metabolism and transport, resulting in rhythmic activation of signaling pathways through modification of the distribution of proteins in lipid rafts at the plasma membrane. We show that rhythmic lipid content is disturbed in the liver of *Scp2* KO mice, which also present a perturbed rhythmic activation of several signaling pathways including PPARα, SREBP, LRH-1, and TORC1. Interestingly, it appeared that these perturbed activated pathways feed back upon the molecular clock itself and delay rhythmic genes expression in a time-specific manner, as well as the circadian period of *Scp2* KO animals. Increasing evidences showed that lipid metabolism and accumulation in mouse liver follow a diurnal rhythm controlled by feeding and the circadian clock^28^, and we report here that these rhythmic lipid metabolism and transport in turn affect activation of signaling pathways and participate to the global rhythmic transcriptome of the mouse liver.

## Results

### SCP2 diurnal expression is regulated at the post-translational level

**Figure 1:**
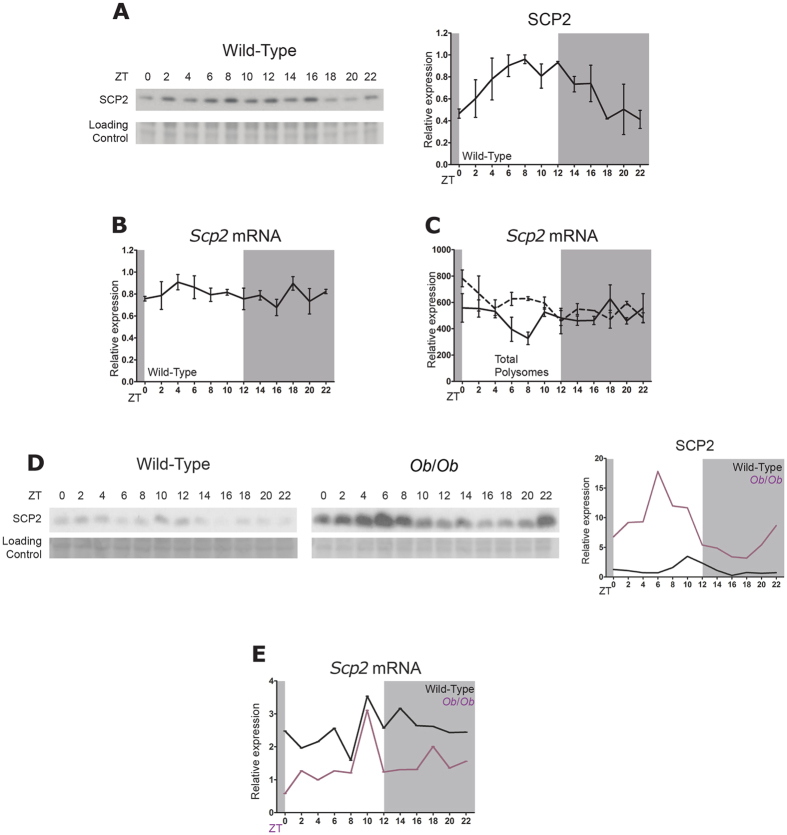
Diurnal accumulation of Sterol Carrier Protein 2. The Zeitgeber Times (ZT), with ZT0: lights on, ZT12: lights off, at which the animals were sacrificed, are indicated on each panel. Night time restricted feeding (NF) or *ad libitum* feeding (AL) conditions are indicated in each individual cases. For all the panels, data for each time point are Mean ± SEM obtained from three independent animals. (**A,D**) Temporal accumulation of SCP2 in WT (NF) (**A**), *Ob/Ob* (AL) (**D**, purple line) and control (AL) (**D**, black line) mouse liver. Representative Western blots were realized on total liver extracts. Naphtol blue black staining of the membranes was used as a loading control. Each graph corresponds to the mean densitometric values of the associated western blots, normalized to the loading control. (**B,E**) Temporal mRNA accumulation of *Scp2* mRNA in WT (NF) (**B**), *Ob/Ob* (AL) (**E**, purple line) and control (AL) (**E**, black line) mouse liver. (**C**) Temporal localization of *Scp2* mRNA in the polysomal fraction of WT mouse liver (AL). Data are extracted from^7^.

Diurnal expression of SCP2 was originally described in rat liver in 1984^20^ and was recently confirmed by proteomic analysis in mouse liver^29^. Post-translational regulation has been suggested as neither *Scp2* mRNA nor its translation appeared rhythmic^21^. We confirmed the rhythmic expression of SCP2 in mouse liver with a maximum of expression around Zeitgeber Time (ZT) 9; with ZT0, lights on and ZT12, lights off (Fig. 1A and Table S1). As previously described, this rhythmic accumulation is not due to the rhythmic accumulation of *Scp2* mRNA (Fig. 1B) or its translation evaluated by the presence of the mRNA in the polysomes (Fig. 1C). Non-characterized post-translational modifications of SCP2, and more particularly degradation, are probably involved in the rhythmic expression of SCP2, as suggested by the numerous ubiquitylation sites characterized on this protein in mouse liver^30^. Moreover, SCP2 rhythmic expression presents a very similar profile to that of liver Triacylglyceride (TAG)^28^, suggesting a potential stabilization of SCP2 by TAG. This observation is consolidated by the fact that *Ob*/*Ob* mice, which present high liver TAG accumulation^31^, also present strongly increased SCP2 liver accumulation despite a decreased expression at the mRNA level (Fig. 1D,E).

### SCP2 influences circadian physiology and diurnal lipid metabolism

**Figure 2:**
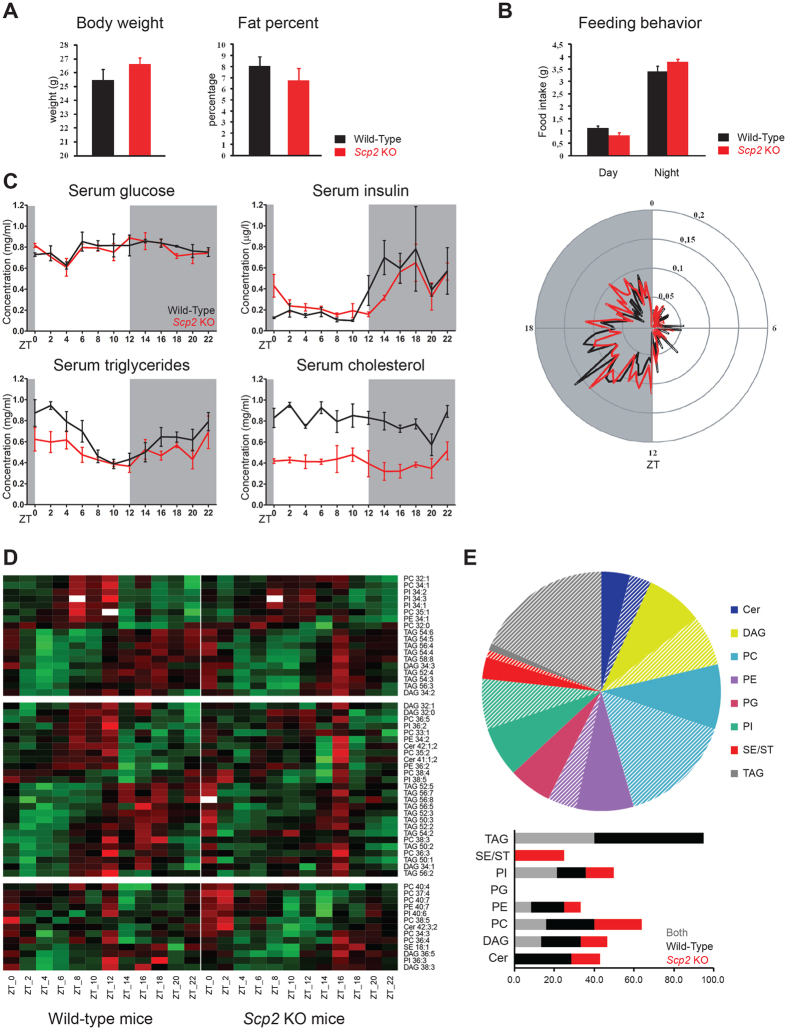
Perturbed metabolism in *Scp2* KO mice. The Zeitgeber Time (ZT) at which the animals were sacrificed is indicated on each panel. (**A**) Body weight (left panel) and fat percentage (right panel) measurements in *Scp2* KO (red lines) and WT (black lines) mouse at ZT3 (AL). Data are Mean ± SEM obtained from five independent animals. (**B**) The amount of food consumed during the light and dark phases is represented in the upper graph and the radar plot (lower panel) shows the diurnal food consumption of the *Scp2* KO (red lines) and WT (black lines) mouse fed AL for five days. Data are Mean ± SEM obtained from five independent animals. (**C**) Temporal serum concentration of glucose, insulin, triglycerides, and cholesterol in *Scp2* KO (red lines) and WT (black lines) mouse (NF). Data are Mean ± SEM obtained from three independent animals. (**D**) Heatmap of the abundance of lipid species in *Scp2* KO (right panels) and WT (left panels) liver (NF). First panel represents lipid species rhythmic in both animals, the second panel species rhythmic only in WT, and the last panel lipid rhythmic only in KO. Standardized relative abundance of each species is indicated in red (high) and green (low). Data are Mean obtained from two independent animals. (**E**) Upper panel: Pie chart representing for each lipid family the proportion of species that are cycling (hatching-colored) and non-cycling (full-colored) in at least one condition (WT, KO, or both). Lower panel: Proportions of cycling lipid species in *Scp2* KO (red), WT (black), or both (grey) mouse liver.

Because SCP2 plays an important role in liver lipid metabolism and transport^25^, we investigated general diurnal metabolic parameters in *Scp2* locus deficient mice. As shown in Fig. 2A, these mice present similar body weight and fat percent compared to controls. In addition, their feeding behavior under light-dark condition and *ad libitum* food access is similar to the one of WT controls (Fig. 2B). However, to avoid potential effect of feeding, mice were fed only during the night throughout the four days before experiment. In this condition, diurnal serum glucose concentration is identical between WT and KO animals, whereas diurnal insulin concentration presents only a mild delay (Fig. 2C and Table S1). However, diurnal serum TAG and cholesterol concentrations are significantly decreased and TAG concentrations present a lower amplitude in KO compared to WT. It strongly suggests that diurnal lipid metabolism is perturbed in *Scp2* KO mice. To gain insight into this perturbed lipid metabolism in the liver, we analyzed the diurnal concentration of lipid species in WT and KO mice. Lipidomic analysis revealed that several lipid species present different levels between WT and KO mice. However, no clear tendencies can be observed except for Cholesterol (ST), and Sterols Esters (SE) that presented, as previously reported^25^,^32^, decreased levels in KO liver, as in serum (Table S2, Fig. S1A). More specifically, diurnal liver Cholesterol levels are low throughout the time and present a decreased amplitude, mainly during the day (Fig. S1B, Table S1).

Several lipid species identified and quantified in both mice present a rhythmic pattern, in agreement with the previously published data (Fig. 2D). However, our statistical analysis reveals a higher proportion of rhythmic species (55% of lipid species are rhythmic in at least one condition, 42% in WT and 30% in KO, compared to the 17% described in^28^), potentially because of the higher sampling frequency (Table S2). Our data suggest that TAG and Diacylglyceride (DAG) generally reach their maximum accumulation during the day while Ceramide (Cer) and Phospholipids (PL) such as Phosphatidylcholine (PC), Phosphatidylethanolamin (PE), and Phosphatidylinositol (PI) reach their maximum during the night (Fig. 2D). Among these families of lipids, most of TAG (95%), around 40% of DAG, Cer, PC, PE, and PI, and no ST or Phosphatidylglycerol (PG) are rhythmic in WT mouse liver (Fig. 2E, upper panel). As mentioned, less species are rhythmic in *Scp2* KO: while 17% of lipids are rhythmic in both WT and KO mice, 25 and 13% are rhythmic only in WT and KO, respectively (Fig. 2E; lower panel). Rhythmic lipid species in both WT and KO liver present a statistically significant 20% decrease in their amplitude (p ≤ 0.05) (Fig. S1C). While most of lipid families contain few species that are rhythmic only in WT or KO, TAG is the only one that did not present species specifically rhythmic only in KO. As a consequence, while 95% of the TAG are rhythmic in WT mice liver, only 40% are rhythmic in KO. These TAG thus present a rhythmic accumulation with a maximum in the beginning of the day, just before SCP2 maximum of accumulation (Fig. S1D). Altogether, these results confirm that SCP2 plays a pivotal role in the rhythmic metabolism and transport of lipids in mouse liver and, as a consequence, this phenomenon is perturbed in *Scp2* KO animals.

### Mouse liver rhythmic transcriptome is dependent on the SCP2 regulated rhythmic lipid transport

**Figure 3:**
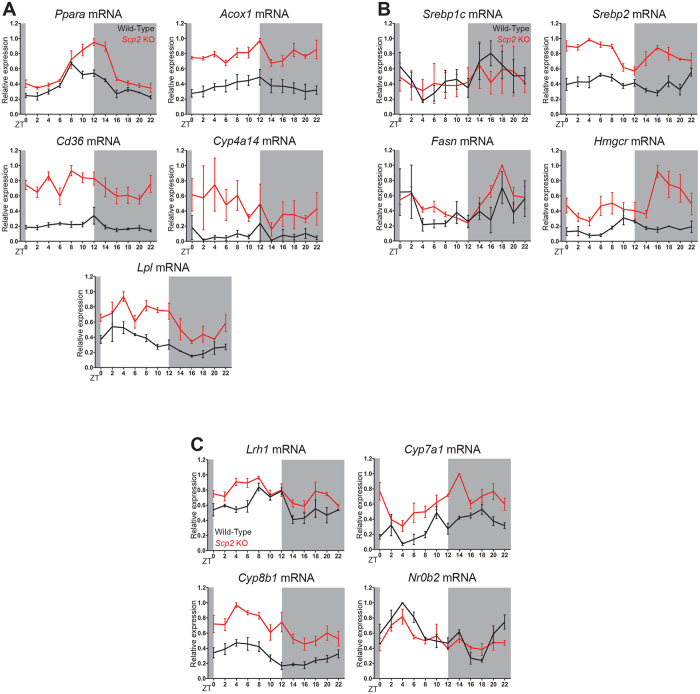
Alteration of the activation of lipids regulated pathways in *Scp2* KO mice. The Zeitgeber Times (ZT) at which the animals were sacrificed are indicated on each panel. All the experiments have been conducted under NF conditions. For all the panels, data for each time point are Mean ± SEM obtained from three independent animals. (**A**) Temporal mRNA expression of *Pparα* and its regulated genes *Acox1*, *Cd36, Cyp4a14* and *Lpl* in *Scp2* KO (red line) and WT (black line) mouse liver. (**B**) Temporal mRNA expression of *Srebp1c* and *Srebp2*, and their regulated genes *Fasn* and *Hmgcr* in *Scp2* KO (red line) and WT (black line) mouse liver. (**C**) Temporal mRNA expression of *Lrh1* and its regulated genes *Cyp7a1*, *Cyp8b1* and *Nr0b2* in *Scp2* KO (red line) and WT (black line) mouse liver.

As discussed in the introduction, many transcription factors and signaling pathways are regulated by lipids and we thus expected that several of them presented a perturbed activity in KO mice. In order to better characterize the impact of the disturbed rhythmic lipid accumulation and metabolism in *Scp2* KO mice liver, rhythmic gene expression was studied by microarray analysis of mRNA harvested every two hours during three consecutive days. Among the genes differentially expressed in WT and KO mice liver, a first analysis of gene expression without consideration of time suggested that several signaling pathways regulated by lipids present an altered activation in KO animals (Table S3). For example, average expression of genes known to be affected by changes of PPARα activity is increased in KO mice. This nuclear receptor directly binds to lipid molecules and activates its target genes involved in different physiological pathways including lipid metabolism^14^. We have previously shown that *Ppar*α transcription and activity are under the control of the circadian clock through direct transcriptional control and synthesis of its ligands, mainly fatty acids (FA)^18^. PPARα is a model of the pathways that could be deregulated in *Scp2* KO mice since availability of these ligands, through control of their transport and synthesis, is potentially under the control of SCP2. As shown in Fig. 3A, expression of genes known to be regulated by PPARα^14^ like Cytochrome P450, family 4, subfamily a, polypeptide 14 (*Cyp4a14*), peroxisomal Acyl-coenzyme A Oxidase 1(*Acox1*), LipoProtein Lipase (*Lpl*), and Cluster of Differentiation 36 (*Cd36*) increased throughout the diurnal cycle, indicating indeed an overall increased activation of the PPARα pathway. This is in accordance with the previously described proliferation of peroxisomes^25^ and activation of PPARα^33^ in the liver of *Scp2* KO mice. Interestingly, *Ppar*α expression is also increased and delayed, probably as a consequence of the regulation of the *Ppar*α promoter by PPARα itself 34.

SREBP is another lipid regulated transcription factor: SREBP is an ER membrane bound protein that, under low sterol conditions, translocates to the Golgi to be cleaved. The remaining peptide is released and migrates to the nucleus, where it activates the transcription of genes coding for enzymes involved in cholesterol and fatty acid metabolism^17^. As shown in Fig. 3B, expression of the genes known to be affected by SREBP activity like 3-Hydroxy-3-MethylGlutaryl-CoA Reductase (*Hmgcr*) and Fatty Acid Synthase (*Fasn*) is upregulated in KO, especially *Hmgcr*, in agreement with the decreased ST concentration in the liver of KO mice (Fig. S2A). Interestingly, while *Fasn* is mostly regulated by SREBP1, *Hmgcr* is regulated by both SREBP isoforms^35^, suggesting a prominent activation of SREBP2 in *Scp2* KO mice. Accordingly, *Srebp2* diurnal expression is upregulated at the transcriptional level in *Scp2* KO mice. This activation of the SREBP pathway is likely a consequence of the observed low Cholesterol and SE levels in the serum and liver of *Scp2* KO, in order to compensate this low concentration (Fig. S1A,B).

LRH-1 is another lipid-binding nuclear receptor which binds to PL to activate genes involved in lipid and glucose metabolisms^15^. PL have been demonstrated as major SCP2 transported lipids^23^ and present an altered rhythmic accumulation in *Scp2* KO mice (Fig. 2). Expression of the genes known to be affected by LRH-1 activity like Cytochrome P450, family 7, subfamily a, polypeptide 1(*Cyp7a1*), Cytochrome P450, family 8, subfamily b, polypeptide 1 (*Cyp8b1*), and Nuclear Receptor subfamily 0, group B, member 2 (*Nr0b2*), as well as *Lrh1* itself, is increased and/or presented a reduction in their diurnal amplitude (Fig. 3C), demonstrating a moderate impact of SCP2 on rhythmic activation of LRH-1 regulated genes. Nevertheless, genes regulated by the oxysterol binding nuclear receptor LXR, which also play an important role in lipid metabolism^16^ and whose activity has been shown to be regulated by the circadian clock^19^, present only a moderate delay in their rhythmic expression (Fig. S2A and Table S1).

**Figure 4:**
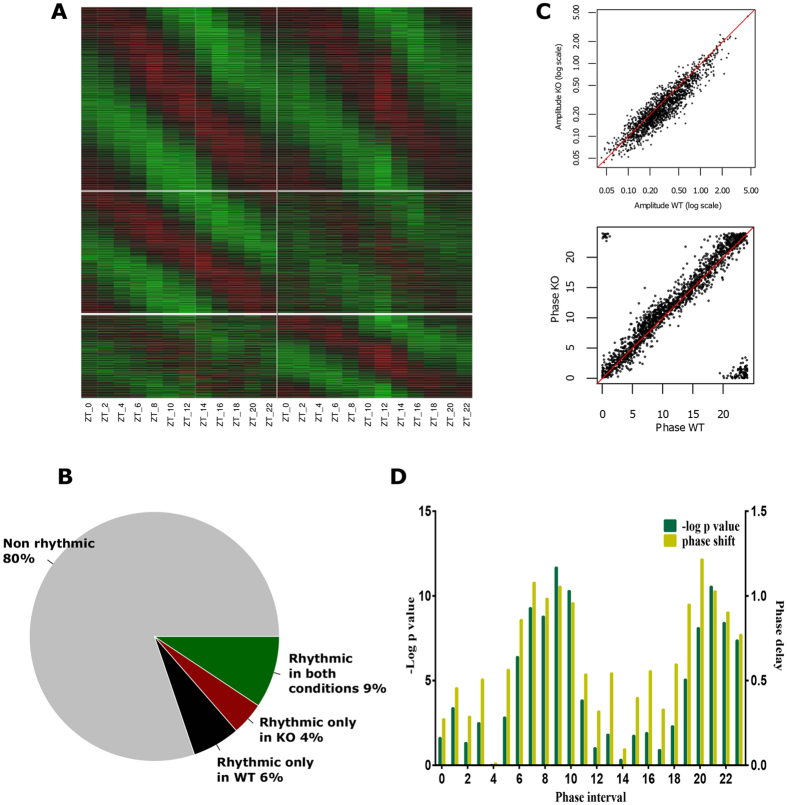
Consequence of *Scp2* deletion on rhythmic liver transcriptome. The Zeitgeber Time (ZT) at which the animals were sacrificed is indicated on each panel. All the experiments have been conducted under NF conditions. (**A**) Heatmap of the cycling and non-cycling genes in *Scp2* KO (right panels) and WT mouse (left panels) liver. First panel represents mRNA probes rhythmic in both animals, the second panel probes rhythmic only in WT, and the last panel probes rhythmic only in KO. Standardized relative expression is indicated in red (high) and green (low). Data are Mean obtained from three independent animals. (**B**) Pie chart representing the proportions of non-cycling (grey) and cycling mRNA probes in *Scp2* KO (red), WT (black), or both (green) mouse liver. (**C**) Scatter plots representing the distribution of amplitudes (upper panel) and phases (lower panel) of rhythmic probes in *Scp2* KO and WT mouse liver. (**D**) Representation of phase delay (hours) of rhythmic mRNA probes observed in *Scp2* KO mouse liver compared to WT (yellow, right y axis) and their significance represented by the -Log of the t-test p-value of the hourly phase distribution of each probes between WT and KO mice (green, left axis).

Specific analysis of the rhythmic transcripts (Fig. 4A and Table S3) shows that while 19% of them are rhythmic in WT or KO, 15% are rhythmic in WT and only 13% in KO. As for lipids, it appears that fewer genes are rhythmic in *Scp2* KO: while 9% of genes are rhythmic in both WT and KO, 6 and 4% are rhythmic only in WT and KO, respectively (Fig. 4B). Genes that present a rhythmic mRNA accumulation in both genotypes present a statistically significant 16% decrease in their amplitude in KO (p ≤ 1.10^−11^), highlighting the strong relationship between rhythmic lipid metabolism and gene expression (Fig. 4C upper panel: more dots are present below the median line). We also observe a significant general phase delay of around 40 min (p ≤ 0.01) between rhythmic genes expressed in WT and KO mice (Fig. 4D lower panel: more dots are present above the median line). However the significance and amplitude of this delay appears phase-dependent, with maximum around ZT 7-10 and ZT 22-23 (Fig. 4C,D). This corresponds to the time when E-box regulated by BMAL1 and ROR elements regulated by REV-ERB/ROR reached their maximum of expression, respectively^19^,^36^. This intriguing observation could suggest that lipid transport perturbation detected in *Scp2* KO mice could feedback on the circadian clock.

### Diurnal activation of signaling pathways is perturbed in *Scp2* KO mice

**Figure 5:**
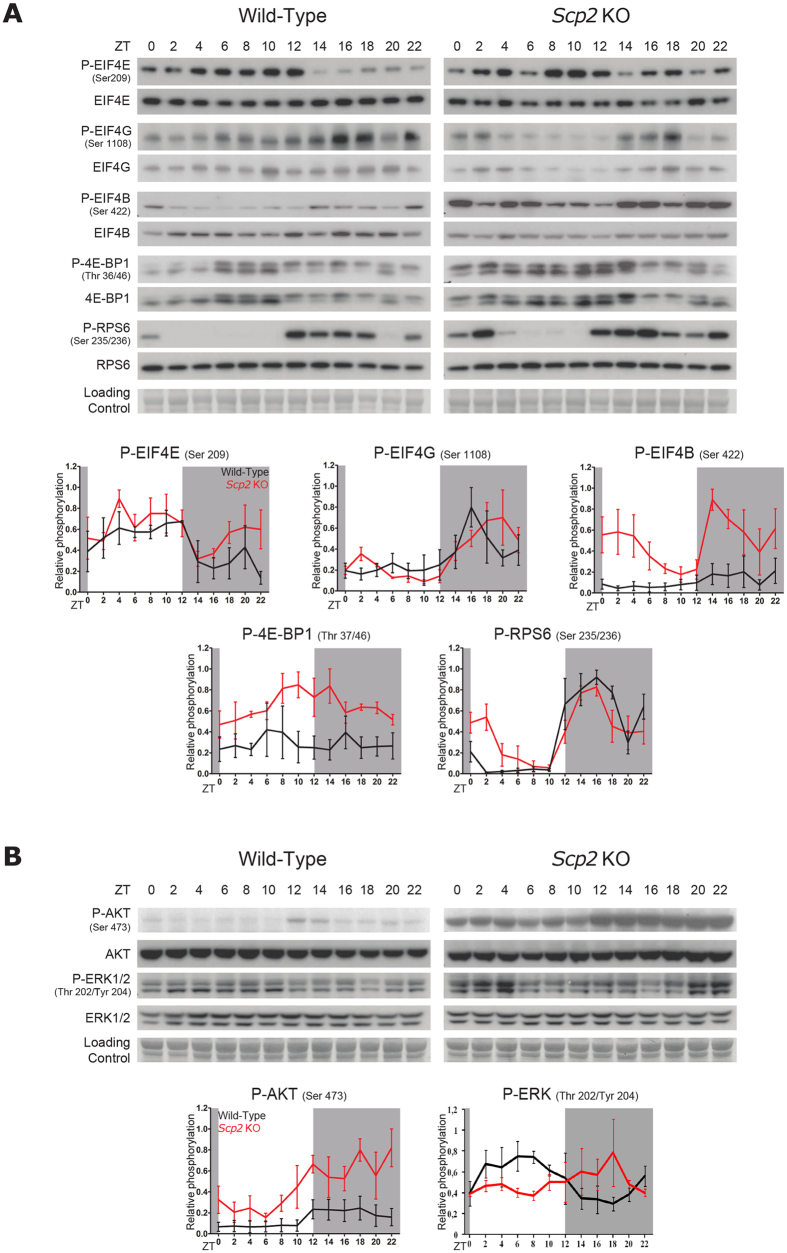
Rhythmic activation of signaling pathways in *Scp2* KO mice. The Zeitgeber Times (ZT) at which the animals were sacrificed are indicated on each panel. All the experiments have been conducted under NF conditions. For all the panels, data for each time point are Mean ± SEM obtained from three independent animals. (**A**) Temporal protein accumulation and phosphorylation of translation initiation factors in *Scp2* KO (right panel) and WT (left panel) mouse liver. Representative Western blots were realized on total liver extracts. Naphtol blue black staining of the membranes was used as a loading control. Each graph corresponds to the mean densitometric values of the associated western blots, normalized to the loading control. (**B**) Temporal protein accumulation and phosphorylation of representative proteins of key signaling pathways involved in the regulation of translation initiation in *Scp2* KO (left panel) and WT (right panel) mouse liver. Representative Western blots were realized on total liver extracts. Naphtol blue black staining of the membranes was used as a loading control. Each graph corresponds to the mean densitometric values of the associated western blots, normalized to the loading control.

We have previously shown that several signaling pathways are rhythmically activated in mouse liver, including the TORC1 pathway^7^. As shown in Fig. 5A, rhythmic activation of the TORC1 pathway is clearly altered in *Scp2* KO mice liver, probably as a consequence of the disturbed activation of the upstream pathways involved in its activation. Indeed, TORC1 activation depends on the activation of several kinases: AMPK inhibits TORC1 through the phosphorylation of Tuberous Sclerosis 2 (TSC2) whereas AKT activates TORC1^37^. TORC1 activation leads to translational activation through the phosphorylation of Eukaryotic translation initiation factor 4E Binding Protein 1 (4E-BP1), Eukaryotic translation Initiation Factor 4G (EIF4G), Eukaryotic translation Initiation Factor 4B (EIF4B), and Ribosomal Protein S6 (RPS6)^38^. In parallel, phosphorylation of Eukaryotic translation Initiation Factor 4E (EIF4E) is mediated through activation of the ERK pathway. In addition to the normal maximum phosphorylation that appeared in the beginning of the night in WT mice^7^, rhythmic phosphorylation of the TORC1 targets RPS6, EIF4B, and EIF4G is perturbed in *Scp2* KO mice with a second peak of phosphorylation in the beginning of the day (ZT2). In addition, we observe an overall increase in phosphorylation level of 4E-BP1 and EIF4B. Differences are observed only at post-translational modifications, with the exception of EIF4G for which the phosphorylated form is unstable, explaining the lower level in the KO^39^, and RPS6, suggesting an interference with ribosome biogenesis (Fig. S3). Concerning the upstream pathways, rhythmic activation of AKT during the night is preserved in *Scp2* KO mice with an overall higher activation throughout the day, and particularly at the day-night transition, explaining in this way the sustained activity of the TORC1 pathway at this period (Fig. 5B). In contrast, rhythmic ERK activation during the day is lost in *Scp2* KO mice with high activation throughout the day, explaining the sustained and somewhat constant phosphorylation of EIF4E. These data converge in a perturbed rhythmic activation of the TORC1 pathway in *Scp2* KO mice, which can lead to perturbed rhythmic ribosome biogenesis, but also lipid metabolism as TORC1 pathway is known to regulate SREBP activity and processing independently of cholesterol concentration^40^,^41^.

We have previously shown that the UPR pathway presents a 12 hour period rhythmic activation orchestrated by the circadian clock^6^. As shown in Fig. S2B, rhythmic maturation of X-box binding protein 1 (*Xbp1*) mRNA and nuclear expression of XBP1 are identical in WT and KO animals, as well as expression of the UPR regulated genes Binding Immunoglobulin Protein (*Bip*) and C/EBP-Homologous Protein (*Chop*). Despite published links between lipid metabolism and UPR activation^8^, this pathway is not affected by SCP2 deficiency.

### Disturbed rhythmic cell signaling feedbacks on the circadian clock

**Figure 6:**
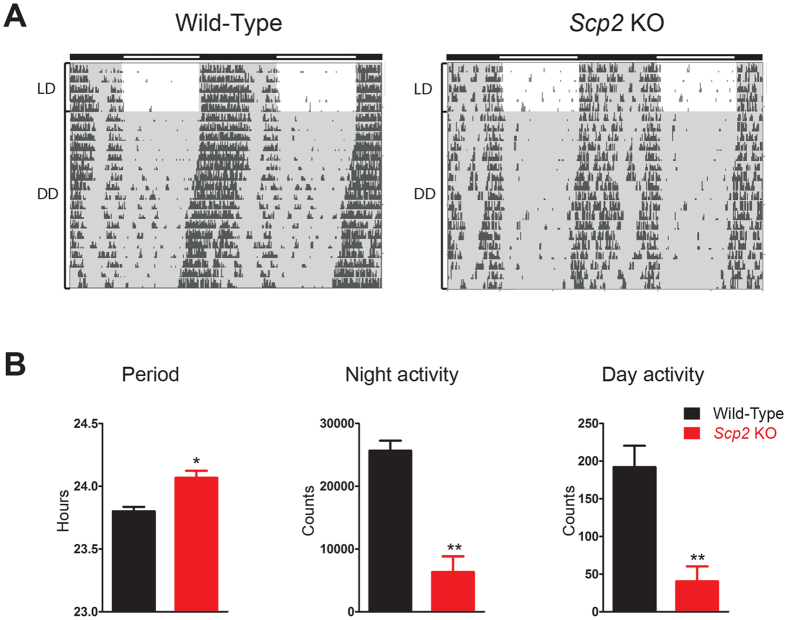
Influence of *Scp2* deletion on the circadian activity. (**A**) Representative circadian locomotor activity (running-wheel) of *Scp2* KO (right panel) and WT (left panel) mice under AL feeding. (**B**) Bar charts representing the free running period (left panel), the night activity (middle panel), and the day activity (right panel) of *Scp2* KO (red bars) and WT mice (black bars). Data are Mean obtained from six independent animals.

Post-translational modifications play a crucial role in the regulation of the circadian clock. Since several of these signaling pathways are disturbed in *Scp2* KO mice, we speculated that it could perturb the circadian clock. To study this possible impact on circadian behavior, we measured the running wheel activity of WT and KO animals in constant darkness. As demonstrated in Fig. 6, *Scp2* KO mice present a significantly longer period by 16 min compared to WT and reduced locomotor activity that did not affect their day-night difference (around 1% activity during the day). Interestingly, the *Scp2* locus has been linked to dystonia and motor neuropathy in human, pathology that can explain this reduced activity^42^.

**Figure 7:**
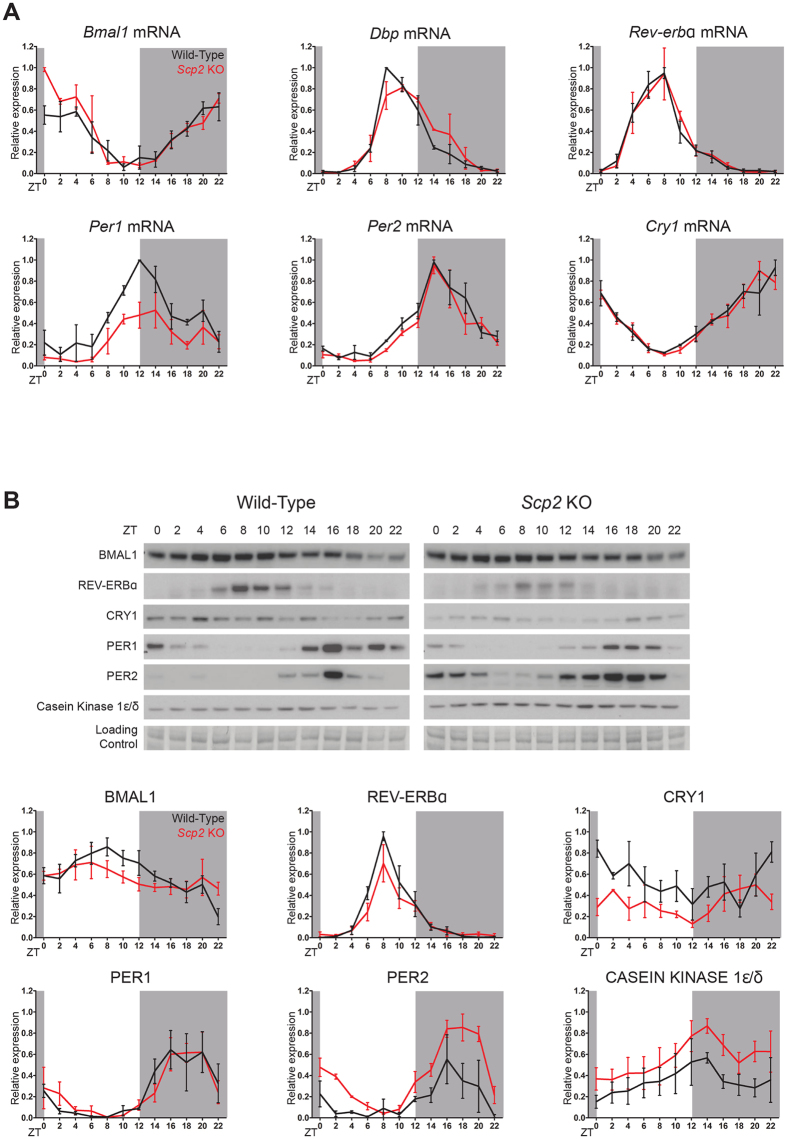
Influence of *Scp2* deletion on the circadian clock. The Zeitgeber Times (ZT) at which the animals were sacrificed are indicated on each panel. All the experiments have been conducted under NF conditions. For all the panels, data for each time point are Mean ± SEM obtained from three independent animals. (**A**) Temporal mRNA accumulation of *Bmal1*, *Dbp*, *Rev-erbα*, *Per1*, *Per2,* and *Cry1* in *Scp2* KO (red line) and WT (black line) mouse liver. (**B**) Temporal protein accumulation of BMAL1, REV-ERBα, CRY1, PER1, PER2 and CK1ε/δ in *Scp2* KO (right panel) and WT (left panel) mouse liver. Representative Western blots were realized on nuclear liver extracts. Naphtol blue black staining of the membranes was used as a loading control. Each graph corresponds to the mean densitometric values of the associated western blots, normalized to the loading control.

We already showed that time-dependent rhythmic genes expression corresponding to E-boxes and ROR-elements described activities seems to be perturbed (Fig. 4D). To study more specifically the possible influence of *Scp2* deletion on circadian clock genes expression, we measured their rhythmic mRNA and protein accumulation in mouse liver. Diurnal expressions of *Bmal1* and *Clock* are slightly delayed and upregulated in KO animals, as the one of their target gene *Dbp* that is slightly delayed (Figs 7A and S4A, and Table S1). Concerning the negative limb of the feedback loop, while *Cry1*, *Cry2*, and *Per2* are not significantly different between *Scp2* KO and WT animals, *Per1* expression is severely downregulated, with reduced amplitude. However, more severe effects are observed at the protein level (Figs 7B and S4B, and Table S1). BMAL1 expression, as well as the one of its target genes REV-ERBα and TEF, presents decreased level and amplitude in KO animals, in addition to an advanced phase (despite an increased mRNA expression for *Tef*  ). While PER1 and CRY2 seem to be moderately affected, PER2 expression is increased and delayed whereas CRY1 expression is decreased. As the accumulation of these proteins is strongly under the control of post-translational modifications^43^, these differences may reflect changes in the rhythmic activation of signaling pathways that control their stability. In this context, Casein Kinase I ε/δ (CK1ε/δ) plays an important function in this process related to its increased rhythmic nuclear accumulation in *Scp2* KO that may explain increased PER2 accumulation.

## Discussion

It has been demonstrated that lipid synthesis and accumulation follow a diurnal rhythm in mouse liver which is controlled by feeding and the circadian clock-regulated lipid metabolism^28^. Here we report that this diurnal lipid transport and metabolism are perturbed in *Scp2* KO mice liver. These KO mice present a deletion of the entire *Scp2* locus, coding for both SCP2 and SCPx. If lipid transport activity of SCP2 is likely involved in this process, it is nevertheless not excluded that SCPx could also contribute to the observed phenotype. Interestingly, SCP2 accumulation follows the one of TAG (Figs 1A and S1D), suggesting a possible stabilization of SCP2 through TAG binding. Protein stabilization through lipid binding is well known for membrane proteins^44^, but has been also described for lipid-bound cytosolic proteins like Perilipins^45^. Considering the modification of rhythmic lipid content in circadian clock mutant mice^28^,^46^, we can speculate that a circadian clock-controlled lipid metabolism adjusts rhythmic SCP2 half-life through formation of stable SCP2-TAG complexes. Although other post-translational mechanisms are possible, this attractive hypothesis constitutes a good example of a rhythmic protein induced by protein-metabolite interaction but further experiments are required to demonstrate this hypothesis. According to the crucial role of post-translational regulations in the establishment of the rhythmic liver proteome^29^, it is not surprising that similar regulation will be described in the near future. In addition, recent evidences showed that TAGs are rhythmic even in the absence of a circadian clock^28^, this rhythm depending mostly on the feeding rhythm. It is conceivable that SCP2, plays a role in that context.

This disturbed lipid accumulation on rhythmic activation of signaling pathways feeds back on the clock itself. Indeed, *Scp2* KO mice present an increased and delayed PER2 accumulation in liver nuclei regulated at the post-translational level, in addition to a lengthening of the free running period. Interestingly, stabilization of PER2 through phosphorylation by CK1ε/δ at Ser659 leads also to period lengthening^47^,^48^. It is thus conceivable that signaling pathways perturbed in *Scp2* KO animals are involved in this period lengthening through the regulation of PER2 degradation. The same hypothesis can explain CRY1 degradation which can be phosphorylated by CK1ε/δ^49^. CRY1 degradation is dependent on its phosphorylation by AMPK^50^, which could also be regulated by lipids^51^. Though there is no clear evidence that CK1ε/δ is regulated by lipids despite a possible regulation by PI^52^, it has been shown that PPARα^53^, LRH-1^54^, AKT^55^, and mTOR^55^,^56^,^57^ pathways interfere with circadian clock regulated gene expression and activity, as well as circadian behavior and physiology. In addition, RORs can bind to hydroxycholesterols which inhibit their transcriptional activity, potentially interacting in this way with E-boxes and ROR-elements transcriptional activity^58^. In parallel, high fat diet has been linked to modification of clock gene expression and circadian behavior^59^. PER2 increased expression observed in *Scp2* KO mice can also be an important player of the observed perturbed lipid metabolism: in addition to its role in the circadian clock, PER2 can bind to several nuclear receptors involved in lipid metabolism^53^ and in this way contributing to the observed phenotype. Taken together, these data suggest that lipid transport and metabolism, which are themselves regulated by the clock, are potentially important regulators of circadian clock function.

Increasing evidences link lipid metabolism and transport to cell signaling via different ways. Lipid metabolism can affect directly the activation of lipid-regulated proteins involved in several signaling pathways. It is for example the case for the nuclear receptors PPARα and LXR in which the rhythmic activation seems to be directly regulated by the rhythmic synthesis of their respective ligands^18^,^19^. Interestingly, these pathways are also regulated by lipid intracellular transport which facilitates direct interaction between these lipids and their ligands^60^. However, in other cases, lipid metabolism acts through the regulation of the cellular localization of the signaling molecules which influence their maturation (for example in the case of SREBP^17^). In addition, lipid metabolism can act via their potential activation by second signaling pathways through their correct localization in a favorable environment or conformation at the plasma membrane, for example in lipid rafts^11^,^12^. As expected, perturbations of lipid metabolism and transport affect also this pathway in all conditions^61^,^62^,^63^. Some interconnections between these two pathways are possible. For example, LXR-regulated cholesterol metabolism disturbs AKT activation through perturbation of its localization in lipid rafts^64^. Nevertheless, no rhythmic activation of signaling pathway by this mechanism has been reported yet. Our results demonstrate that this rhythmic activation of signaling pathways is perturbed in the liver of *Scp2* KO animals.

However, involvement of other lipid transporters in this mechanism is not excluded. For example, the Niemann-Pick C1 (NPC1) protein, which is specifically involved in intracellular cholesterol transporter, is involved in the regulation of several signaling pathways^65^. Indeed, NPC1 inhibition interferes with lipid raft formation and, as a consequence, activation of the LXR and SREBP^66^, PI3K/AKT^67^, Insulin Receptor^68^, and MAPK^69^ pathways. Interestingly, we report here that NPC1 expression is rhythmic at both mRNA and protein levels, and regulated by the circadian clock in mouse liver through transcriptional and post-transcriptional regulations (Fig. S5), suggesting a potential role of NPC1 in diurnal activation of signaling pathways. Further experiments are required to characterize the importance of these two, or additional, lipid transporters in rhythmic activation of signaling pathways.

## Methods

### Animal experiments

All animal studies were conducted in accordance with our internal ethics committee for animal experimentation and were approved by the veterinary office of the Canton of Vaud (Authorization VD2720). Eight-week-old male C57Bl/6J and C57Bl/6J-ob (*Ob*/*Ob*) mice were purchased from Charles River Laboratory (L’Arbresle, France). *Scp2* KO mice backcrossed into the C57Bl/6 background have been previously described^25^ and have been acquired from Jackson Laboratory, as well as their corresponding C57Bl/6J control mice. *Bmal1*^7^ and *Cry1*/*Cry2*^70^ KO mice have been already described. In all experiments, male mice between 10 and 12 weeks of age are used. Unless noted otherwise, mice were maintained under standard animal housing conditions, with free access to food and water and in 12 hours light/12 hours dark cycles. However, for all experiments, animals were fed only at night during 4 days before the sacrifice to reduce effects of feeding rhythm. Individual mice were sacrificed and samples collected every 2 hours during a 24 hours period.

The running-wheel activity has been monitored as previously described^71^. Briefly, the mice were housed individually in cages equipped with a running-wheel (Phenome Technologies). The activity has been measured during 5 days in light-dark cycles followed by 18 days in constant darkness. Data were acquired and analysed with the Clocklab software (Actimetrics). Feeding behavior has been monitored with the “Feeding and Drinking Monitor” system from TSE Systems. Briefly, 10 weeks aged mice were individually placed in equipped cages with free access to food and water, in a 12 hours light/12 hours dark environment. Measurements of food intake were performed at 15 min resolution throughout five consecutive days. The fat percentage has been determined by using the Nuclear Magnetic Resonance technology using an EchoMRI-700 analyzer (EchoMRI^TM^). At ZT3, 10 weeks aged mice were weighted and underwent EchoMRI.

### RNA extraction and analysis

Liver RNA were extracted and analysed by real-time quantitative RT-PCR, mostly as previously described^72^. Microarray analyses are described in Supplemental Information.

### Protein extractions and analysis

Nuclear and total proteins were extracted and analysed mostly as described^72^. Experimental details are given in Supplemental Information.

### Serum chemistry analysis

Blood samples are collected every 2 hours and sera are obtained after a centrifugation of 10 min at 10 000 rpm at room temperature. Sera are kept at −80 °C until analysis. Insulin, glucose, cholesterol and triglycerides are respectively measured accordingly with the protocols of the Mouse Insulin ELISA kit (Mercodia), and the Glucose, Cholesterol, Triglycerides LabAssay kits (Wako).

### Lipid Extraction and analysis

Approximately 10 mg of pulverized liver tissue was homogenized in 1 ml of ammonium bicarbonate buffer (concentration: 150 mM of ammonium bicarbonate in water) using Tissue Lyser at a speed of 25 Hz for 2.5 min. Supernatant was removed into new glass tube on ice. 100 μl of the homogenate was collected and further diluted with 80 μl of Ammonium bicarbonate buffer using Hamilton Robot and 810 μl of MTBE /Methanol (7/2 v/v) containing internal standard was added to this mixture. The internal standard mixture contained: LPG 17:1, LPA 17:0, PC 17:0/17:0, PS 17:0/17:0, PG 17:0/17:0, PA 17:0/17:0, LPI 13:0, LPS 13:0, Chol D6, DAG 17:0/17:0, TAG 17:0/17:0/17:0, Cer 18:1;2/17:0, SM 18:1;2/12:0, LPC 12:0, LPE 17:1, PE 17:0/17:0, CE 20:0, PI 16:0/16:0. Solution was mixed at 700 rpm, 15 min at 4 °C using a ThermoMixer C and then centrifugated at 3000 RCF for 5 min. 100 μl of the organic phase was transferred to a 96-well plate, and dried in a speed vacuum concentrator. Lipid extract was reconstituted in 40 μl of 7.5 mM ammonium acetate in chloroform/methanol/propanol (1:2:4, V/V/V). All liquid handling steps were performed using Hamilton STAR robotic platform.

For MS data acquisition**, s**amples were analyzed by direct infusion in a QExactive mass spectrometer (Thermo Fisher Scientific) equipped with a TriVersa NanoMate ion source (Advion Biosciences). Samples were acquired in both polarity modes in a single acquisition at R_m/z=200_ = 140000. All data was analyzed with in-house developed lipid identification software based on LipidXplorer. Data post-processing and normalization were performed on an in-house developed R based package.

### mRNA and lipid rhythmicity analysis

In order to assess rhythmicity, we used harmonic regression with a focus on 24 hours periodicity. We performed mutilinear regression on log transform values:


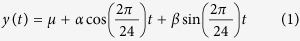


with:


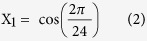


and


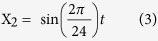


the explanatory variables, y the dependent variable, μ the mean of the data while a and b are respectively the coefficient of cosine and sine function with a period of 24 hours. All relevant quantities as peak-through amplitude, relative amplitude, and tangent of the phase can be calculated explicitly. Statistical significance against the null hypothesis H_0_ that a = 0 and b = 0 is achieved by F-test for linear regression. We corrected the p-value for False Discovery Rate (FDR) using Benjamini–Hochberg method^73^. For lipid analyses, for each sample the data were normalized by their respective tissue mass. Lipids species with more than ten null values were discarded. Null values were considered as missing time points for the multilinear regression.

### Cosinor analysis of rhythmic parameters

The rhythmic characteristics of the expression of each gene or protein were assessed by a Cosinor analysis^74^. This method characterizes a rhythm by the parameters of the fitted cosine function best approximating the data. A period of 24 h was a priori considered. The rhythm characteristics estimated by this linear least squares method include the mesor (rhythm-adjusted mean), the double amplitude (difference between minimum and maximum of fitted cosine function), and the acrophase (time of maximum in fitted cosine function). A rhythm was detected if the null hypothesis was rejected with *P *< 0.05. In such a case, the 95% confidence limits of each parameter were computed. The Cosinor 2.3 software used in this study has been elaborated by the Circadian Rhythm Laboratory at University of South Carolina and is freely available at this address: http://www.circadian.org/softwar.html.

## Additional Information

**How to cite this article**: Jouffe, C. *et al.* Perturbed rhythmic activation of signaling pathways in mice deficient for Sterol Carrier Protein 2-dependent diurnal lipid transport and metabolism. *Sci. Rep.* **6**, 24631; doi: 10.1038/srep24631 (2016).

## Supplementary Material

Supplementary Information

Supplementary Table S1

Supplementary Table S2

Supplementary Table S3
