# Supplementary material for: Perturbed rhythmic activation of signaling pathways in mice deficient for Sterol Carrier Protein 2-dependent diurnal lipid transport and metabolism
Source: Sci Rep. 2016 Apr 21;6:24631. doi: 10.1038/srep24631 (PMC4838911; doi:10.1038/srep24631)
Supplement: Supplementary Information [file srep24631-s1.pdf]

Perturbed rhythmic activation of signaling pathways in mice deficient for Sterol Carrier Protein 2-dependent diurnal lipid transport and metabolism

Céline Jouffe, Cédric Gobet, Eva Martin, Sylviane Métairon, Delphine Morin-Rivron, Mojgan Masoodi, and Frédéric Gachon

Supplemental Information

Figure S1: SCP2 influences lipid composition in mouse liver

Figure S2: Regulation of LXR and UPR pathways in *Scp2* KO mice

Figure S3: Temporal expression of translation initiation complex factors

Figure S4: The circadian clock is influenced by *Scp2* deletion

Figure S5: Rhythmic expression of NPC1 is circadian clock dependent.

Supplemental Tables

Supplemental Experimental Procedures

Supplemental References

Figure S1: SCP2 influences lipid composition in mouse liver

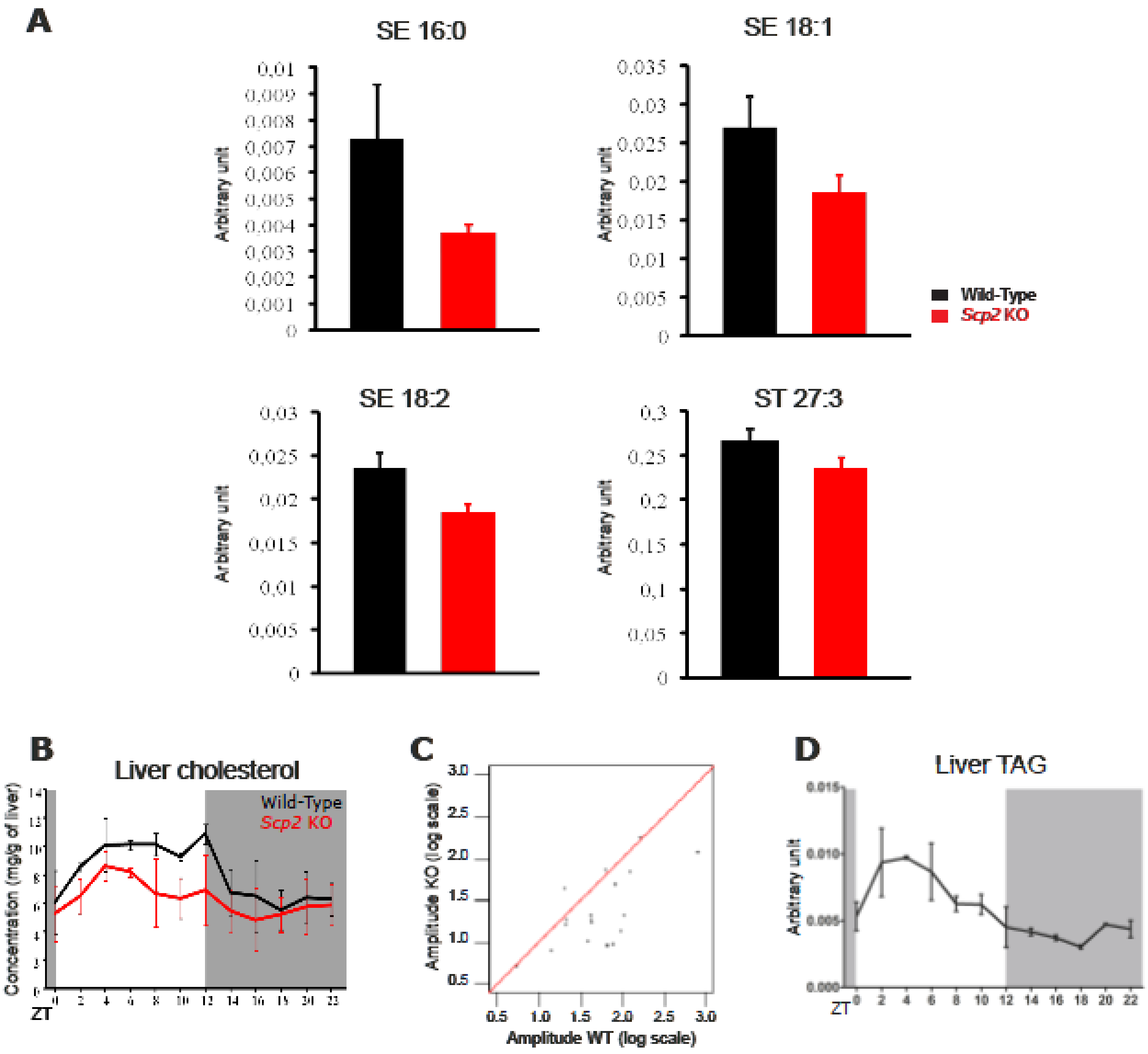

The Zeitgeber Times (ZT) at which the animals were sacrificed is indicated on the panel. All the experiments have been conducted under NF conditions. For each time point, data are Mean ± SEM obtained from two independent animals.

A. Daily amount of accumulated sterol (ST) and sterol ester (SE) species in *Scp2* KO (red bars) and WT (black bars) mouse liver.

B. Rhythmic accumulation of cholesterol in WT (black line) and *Scp2* KO (red line) mouse liver.

C. Scatter plot representing the amplitudes of rhythmic accumulated lipids in *Scp2* KO and WT mouse liver.

D. Rhythmic accumulation of TAG in WT mouse liver.

Figure S2: Regulation of LXR and UPR pathways in *Scp2* KO mice

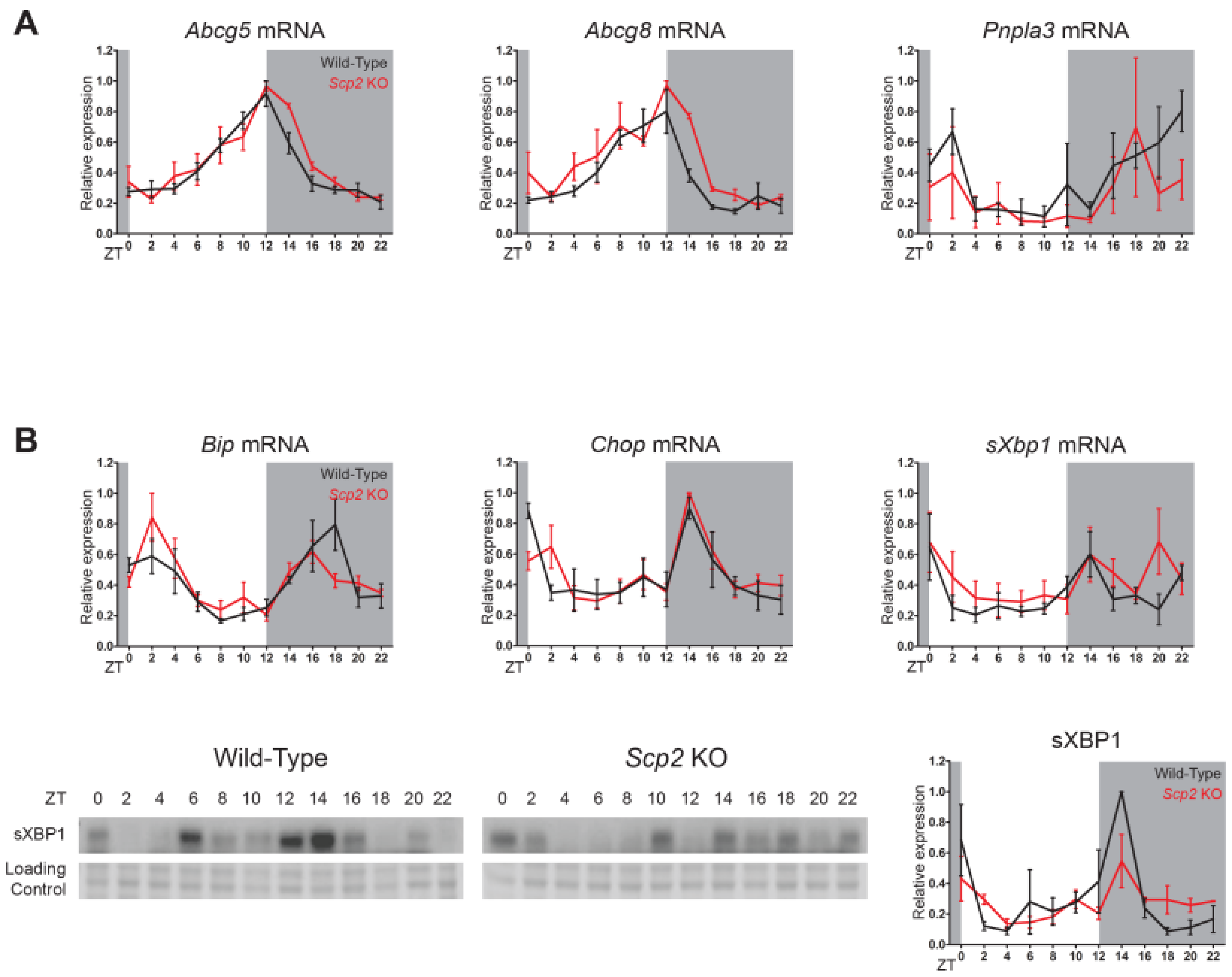

The Zeitgeber Time (ZT) at which the animals were sacrificed is indicated on each panel. All the experiments have been conducted under NF conditions. For all the panels, data for each time point are Mean ± SEM obtained from three independent animals.

A. Temporal mRNA accumulation of LXR regulated genes *Abcg5*, *Abcg8* and *Pnpla3* in *Scp2* KO (red line) and WT (black line) mouse liver.

B. Temporal mRNA accumulation of genes involved in the regulation of the UPR *Bip*, *Chop*, and *sXbp1* in *Scp2* KO (red line) and WT (black line) mouse liver (upper panels). Temporal accumulation of sXBP1 in *Scp2* KO (right panel) and WT (left panel) mouse liver (lower panels). Representative Western blots were realized on nuclear liver extracts. Naphtol blue black staining of the membranes was used as a loading control. The graph corresponds to the

mean densitometric values of the associated western blots, normalized to the loading control. The described 12 hours period activation of UPR is conserved in *Scp2* KO mice (1).

Figure S3: Temporal expression of translation initiation complex factors

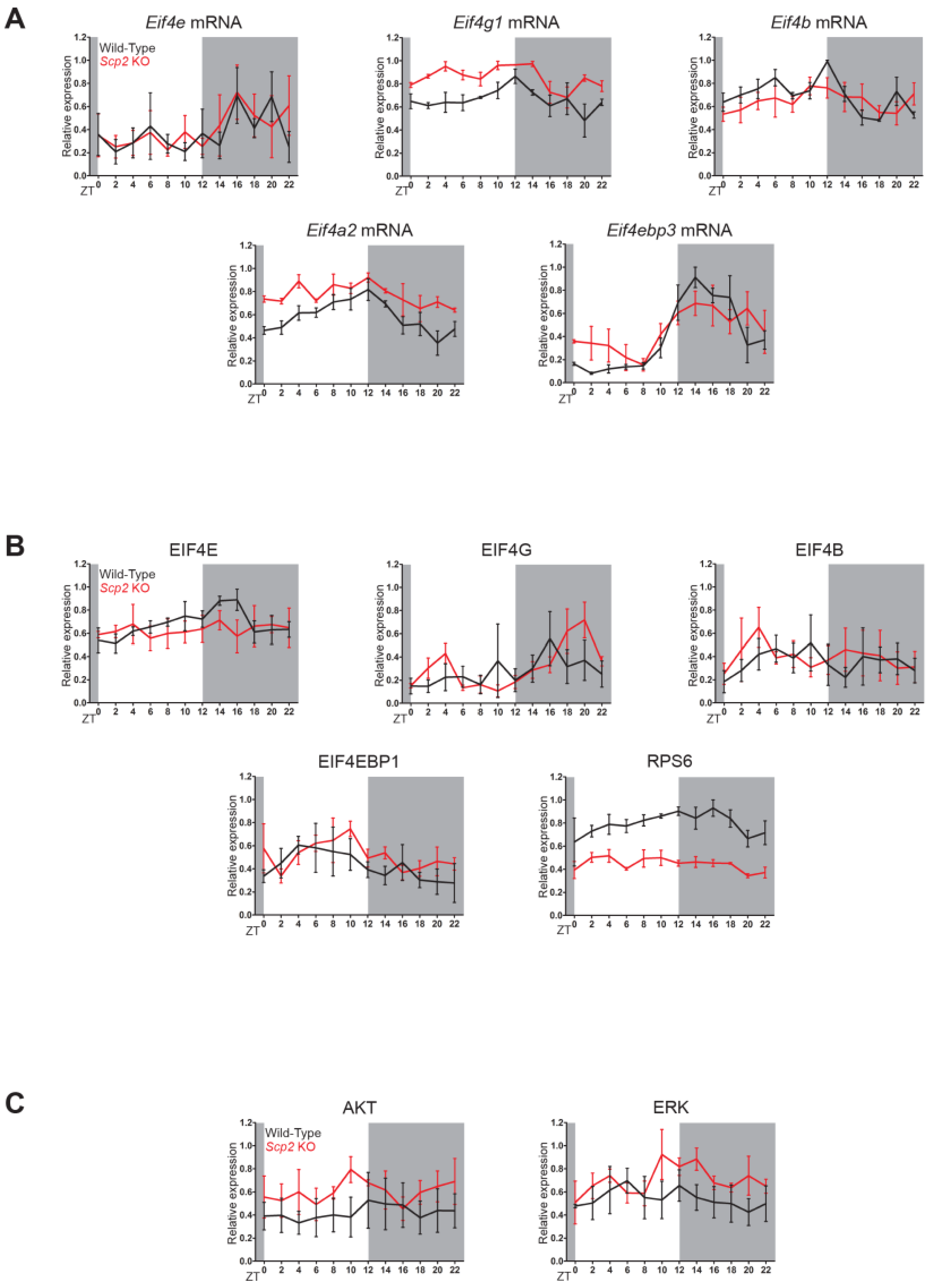

The Zeitgeber Time (ZT) at which the animals were sacrificed is indicated on each panel. All the experiments have been conducted under NF conditions. For all the panels, data for each time point are Mean ± SEM obtained from three independent animals.

A. Temporal mRNA accumulation of translation initiation factors in *Scp2* KO (red line) and WT (black line) mouse liver.

B. Temporal protein accumulation of translation initiation factors in *Scp2* KO (red line) and WT (black line) mouse liver. Each graph corresponds to the densitometric values of the associated western blots, normalized to the loading control.

C. Temporal accumulation of proteins involved in signaling pathways involved in translation initiation in *Scp2* KO (red line) and WT (black line) mouse liver. Each graph corresponds to the densitometric values of the associated western blots, normalized to the loading control.

Figure S4: The circadian clock is influenced by *Scp2* deletion

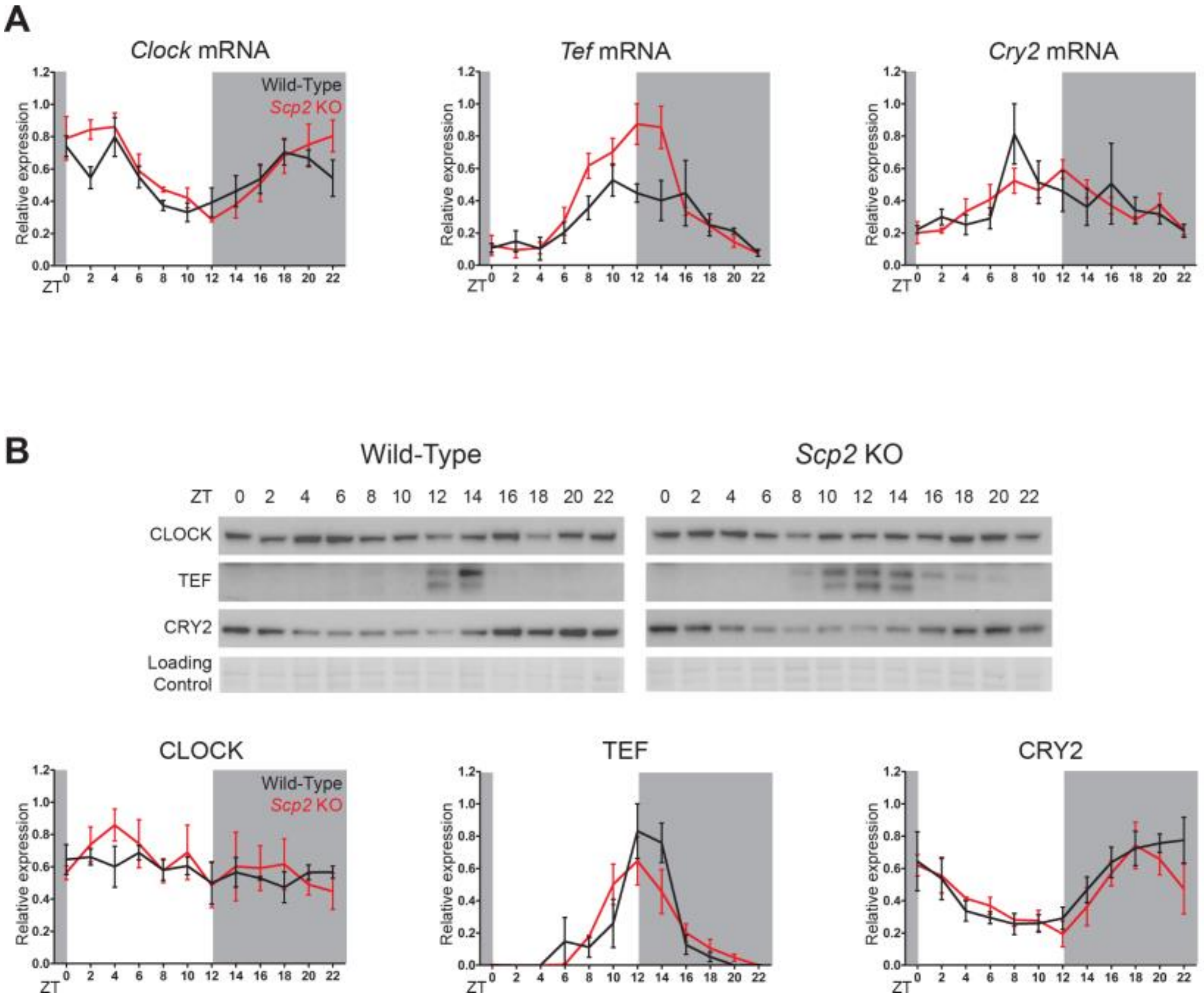

The Zeitgeber Time (ZT) at which the animals were sacrificed is indicated on each panel. All the experiments have been conducted under NF conditions. For all the panels, data for each time point are Mean ± SEM obtained from three independent animals.

A. Temporal mRNA accumulation of *Clock*, *Tef*, and *Cry2* in *Scp2* KO (red line) and WT (black line) mouse liver.

B. Temporal protein accumulation of CLOCK, TEF, and CRY2 in *Scp2* KO (right panel) and WT (left panel) mouse liver. Representative Western blots were realized on nuclear liver extracts. Naphtol blue black staining of the membranes was used as a loading control. Each graph corresponds to the mean densitometric values of the associated western blots, normalized to the loading control.

Figure S5: Rhythmic expression of NPC1 is circadian clock dependent.

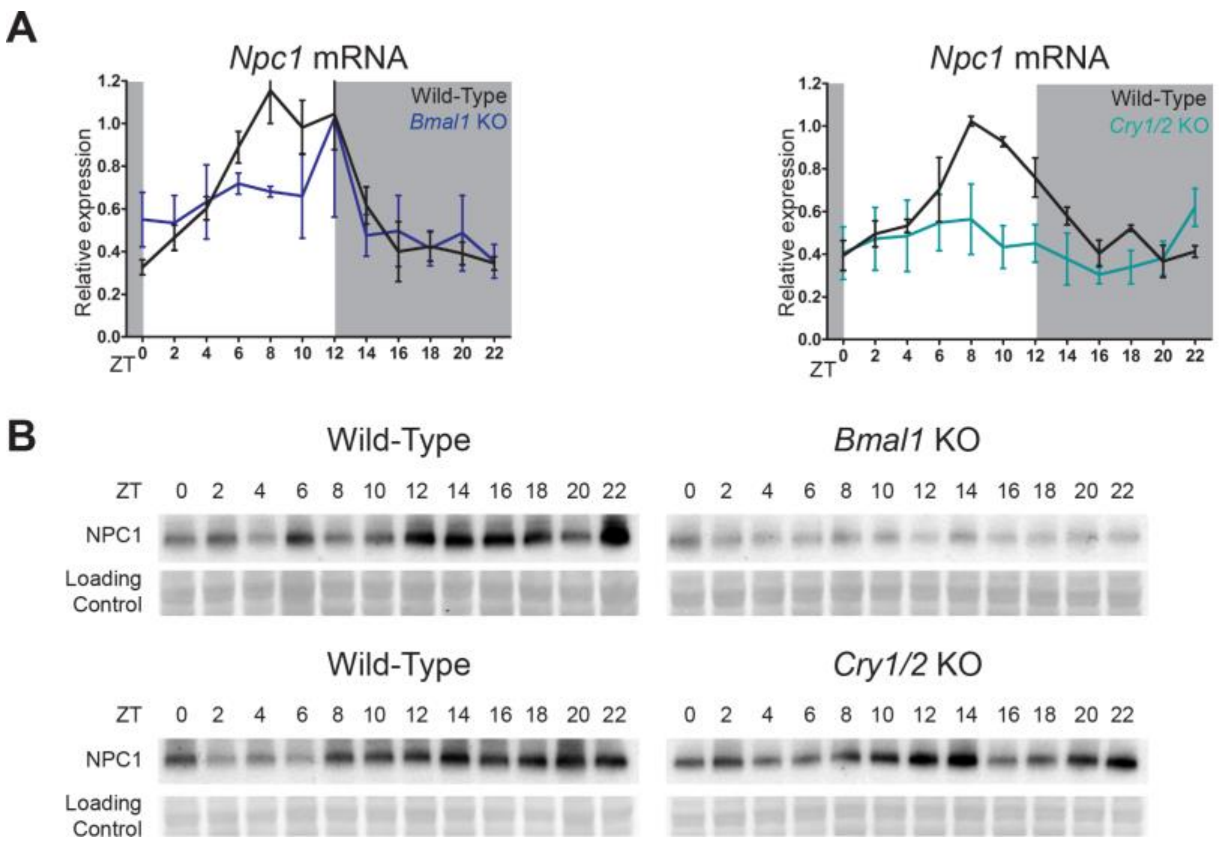

The Zeitgeber Time (ZT) at which the animals were sacrificed is indicated on each panel. All the experiments have been conducted under NF conditions. For all the panels, data for each time point are Mean ± SEM obtained from three independent animals.

A. Liver *Npc1* mRNA accumulation was measured by real-time RT-PCR on liver obtained from arrhythmic *Bmal1* (right panel) and *Cry1*/*Cry2* (left panel) KO mice and their control littermates.

B. Protein accumulation were assessed by western-blot on total extracts from *Bmal1* (upper panel) and *Cry1*/*Cry2* (lower panel) KO mice and their control littermates. Naphtol blue black staining of the membranes was used as a loading control.

Supplemental Tables

Table S1: Cosinor statistical values related to rhythmic mRNA and protein accumulation of genes encoding regulator of signaling pathways and the circadian clock, and to serum and liver metabolites measured in *Scp2*, *Ob/Ob* and WT mice. The rhythmic mRNAs, proteins and metabolites ($p<0,05$) are indicated in red.

Table S2: Liver lipidomic data obtained by Mass Spectrometry experiment in *Scp2* KO and WT mouse liver and the associated analysis of rhythmicity. The experiment has been conducted under NF conditions. The lipid species that present a significant ($p<0,05$) rhythmic accumulation or a different level between WT and KO are indicated in red.

Table S3: Transcriptomic data obtained by microarray experiment in *Scp2* KO and WT mouse liver and the associated analysis of rhythmicity. The experiment has been conducted under NF conditions. Genes that present a significant ($p<0,05$) rhythmic accumulation or a different level between WT and KO are indicated in red.

Table S4: Taqman probes used for real-time PCR (Life technologies)

**Gene**	**Probe reference**
Gapdh	Mm 99999915_g1
Ppara	Mm00440939_m1
Lpl	Mm00434770_m1
Fasn	Mm00662319_m1
Lrh1(*Nr5a2*)	Mm00446088_m1
Cyp7a1	Mm00484150_m1
Cyp8b1	Mm00501637_s1
Nr0b2	Mm00442278_m1
Abcg5	Mm 00446241_m1
Abcg8	Mm 00445980_m1
Pnpla3	Mm 00504420_m1
Eif4e	Mm 00725633_s1
Eif4g1	Mm 00524099_m1
Eif4b	Mm00778003_s1
Eif4a2	Mm 00778003_s1
Eif4ebp3	Mm 01406408_m1

Table S5: Sequences of the primers used for real-time PCR

Gene	Forward primer	Reverse primer
Gapdh	CATGGCCTTCCGTGTTCCTA	CCTGCTCTTCCGTGTTCCTA
Scp2	GGCCTTCTTTCAAGGGAAAC	CTAAGCCCTGACGACGAGAC
Acox1	GGATGGTAGTCCGGAGAACA	AGTCTGGATCGTTCAGAATCAAG
Cd36	GATGACGTGGCAAAGAACAG	TCCTCGGGGTCCTGAGTTAT
Cyp4a14	TCTCTGGCTTTTCTGTACTTTGCTT	CAGAAAGATGAGATGACAGGACACA
Srebp1c	GGAGCCATGGATTGCACATT	GGCCCGGGAAGTCACTGT
Srebp2	GCGTTCTGGAGACCATGGA	ACAAAGTTGCTCTGAAAACAAATCA
Hmgcr	AGCTTGCCCGAATTGTATGTG	TCTGTTGTGAACCATGTGACTTC
Bmal1	GCATTCTTGATCCTTCTTTGGT	CCAAGAAGGTATGGACACAGACAAA
Dbp	CGTGGAGGTGCTTAATGACCTTT	CATGGCCTGGAATGCTTGA
Rev-erbα	CATGGTGCTACTGTGTAAGGTGTGT	CACAGGCGTGCACTCCATAG
Per1	ACCAGCGTGTCATGATGACATAC	CTCTCCCGGTCTTGCTTCAG
Per2	ATGCTCGCCATCCACAAGA	GCGGAATCGAATGGGAGAAT
Cry1	CTGGCGTGGAAGTCATCGT	CTGTCCGCCATTGAGTTCTATG
Bip	GAAAGGATGGTTAATGATGCTGAG	GTCTTCAATGTCCGCATCCTG
Chop	CATACACCACCACACCTGAAAG	CCGTTTCCTAGTTCTTCCTTGC
sXbp1	CTGAGTCCGAATCAGGTGCAG	TGGCCGGGTCTGCTGAGTCCG
Clock	TTGCTCCACGGGAATCCTT	GGAGGGAAAGTGCTCTGTTGTAG
Tef	GCCGAGCTTCGCAAGGA	ACAGGTTACAAGGGCCCGTACT
Cry2	TGTCCCTTCCTGTGTGGAAGA	GCTCCCAGCTTGGCTTGA
Npc1	TGAATGCGGTCTCCTTGGTC	CTCACTGGCTTCCTTTGGTA

Table S6: References of the antibodies used for Western blotting

Protein	Reference	Company
SCP2	HPA027101	Sigma
P-EIF4E (Ser 209)	9741	Cell Signaling Technology
EIF4E	2067	Cell Signaling Technology
P-EIF4G (Ser 1108)	2441	Cell Signaling Technology
EIF4G	2469	Cell Signaling Technology
P-EIF4B (Ser 422)	3591	Cell Signaling Technology
EIF4B	3592	Cell Signaling Technology
P-4EBP1 (Thr 37/46)	2855	Cell Signaling Technology
4EBP1	9644	Cell Signaling Technology
P-RPS6 (Ser 235/236)	2211	Cell Signaling Technology
RPS6	2217	Cell Signaling Technology
P-AKT (Ser 473)	4060	Cell Signaling Technology
AKT	4691	Cell Signaling Technology
P-p44/42 MAPK (Erk1/2) (The 202 / Tyr 204)	4376	Cell Signaling Technology
P44/42 MAPK (Erk1/2)	9102	Cell Signaling Technology
BMAL1	Reference (2)	
REV-ERBα	Reference (2)	
CRY1	Reference (2)	
PER1	Reference (3)	
PER2	Reference (3)	
CASEIN KINASE 1ε/δ	2655	Cell Signaling Technology
sXBP1	Sc-7160	Santa Cruz Biotechnology
CLOCK	Reference (2)	
TEF	Reference (4)	
CRY2	Reference (2)	
NPC1	Ab36983	Abcam

Supplemental Experimental Procedures

Real-Time PCR analysis of mRNA expression

0.5 µg of liver RNA was reverse transcribed using random hexamers and SuperScript® II reverse transcriptase (Life Technologies). The cDNAs equivalent to 20 ng of RNA were PCR amplified in a LightCycler® 480 II System (Roche) using the TaqMan® or the SYBR® Green technologies. References and sequences of the probes are given in the tables. In each case, averages from at least three independent experiments are given, using *Gapdh* mRNA as controls. Probes references are given in tables S4 and S5.

Microarray analysis

For microarray analysis, RNA was quantified using Quant-It Ribogreen assay (LifeTechnologies) on spectramax reader (Molecular Probes) and purity was verified with the Fragment Analyzer (Advanced Analytical Technologies, Inc.). All cRNA targets were synthesized, labeled and purified according to the Illumina TotalPrep-96 RNA amplification protocol. 300 ng of total RNA were used to produce double-stranded cDNA, followed by an in vitro transcription, along with biotin UTP. This method is based on the Eberwine T7 procedure (5). Prior to the hybridization on the arrays, 750 ng of biotin labeled-cRNAs were used to prepare the hybridization mix, which contained control oligonucleotides (such as negative and hybridization controls), hybridization buffer, and water. Then, 15 µl of each hybridization mix were dispensed on the arrays. After an overnight hybridization (16 hours, 58°C), the arrays were washed to remove non-hybridized material, and stained with Streptavidin-Cy3 which bound with biotin. All samples were analyzed on MouseRef-8 V2.0 Expression BeadChips (Illumina, San Diego, CA, USA), which comprise probes to interrogate transcripts. Scanning was performed using the HiScan (Illumina), which provides intensity values for all transcripts, measuring the signal emitted by the Streptavidin-Cy3 conjugates responding to a laser excitation. Signal intensities were extracted and summarized in the BeadStudio software (Illumina). Data were expressed as absolute intensities. Raw data were then preprocessed using Partek software. A log2 transformation was performed, in order to shape the data into a normal distribution, followed by a quantile normalization to equal the distribution of the 72 arrays prior to the statistical analysis. Data are deposited on the Gene Expression Omnibus database under the reference GSE67426.

Nuclear protein extractions and analysis

Liver were homogenized in sucrose homogenization buffer containing 2.2 M sucrose, 15 mM KCl, 2 mM EDTA, 10 mM HEPES (pH7.6), 0.15 mM spermin, 0.5 mM spermidin, 1 mM DTT, and a protease inhibitor cocktail containing 0.5 mM PMSF, 10 µg/ml Aprotinin, 0.7 µg/ml Pepstatin A, and 0.7 µg/ml Leupeptin. Lysates were deposited on a sucrose cushion containing 2.05 M sucrose, 10 % glycerol, 15 mM KCl, 2 mM EDTA, 10 mM HEPES (pH7.6), 0.15 mM spermin, 0.5 mM spermidin, 1 mM DTT, and a protease inhibitor cocktail. Tubes were centrifuged during 45 min at 105 000 g at 4 °C. After ultra-centrifugation, the nucleus pellets were suspended in a nucleus lysis buffer composed of 10 mM HEPES (pH7.6), 100 mM KCl, 0.1 mM EDTA, 10 % Glycérol, 0.15 mM spermin, 0.5 mM spermidin, 0.1 mM NaF, 0.1 mM sodium orthovanadate, 0.1 mM ZnSO4, 1 mM DTT, and the previously described protease inhibitor cocktail. Nuclear extracts were obtained by the addition of an equal volume of NUN buffer composed of 2 M urea, 2 % Nonidet P-40, 600 mM NaCl, 50 mM HEPES (pH7.6), 1 mM DTT and a cocktail of protease inhibitors, and incubation 20 min on ice. After centrifugation during 10 min at 21 000 g, the supernatants were harvested and constitute nuclear extracts.

12.5 µg nuclear extracts were used for western blotting. After migration, proteins were transferred to PVDF membranes and western blotting was realized according to standard procedures. References for the antibodies are given in table S6.

Total protein extractions and analysis

Frozen organs were homogenized in lysis buffer containing 20 mM HEPES (pH 7.6), 100 mM KCl, 0.1 mM EDTA, 1 mM NaF, 1 mM sodium orthovanadate, 1 % Triton X-100, 0.5% Nonidet P-40, 0.15 mM spermin, 0.5 mM spermidin, 1 mM DTT, and the same protease inhibitor cocktail as for nuclear protein extractions. After incubation 30 min on ice, extracts were centrifuged 10 min at 21 000 g and the supernatants were harvested to obtain total extracts.

65 µg of extract was used for western blotting. After migration, proteins were transferred to PVDF membranes and western blotting was realized according to standard procedures. References for the antibodies are given in table S6.

Liver lipid extraction and cholesterol analysis

Frozen liver pieces were heated in an alcoholic KOH solution for 6 hours and precipitated with $MgCl_2$. After centrifugation for 30 minutes at 14 000 rpm, the lipids were collected. Liver cholesterol was measured in accordance with the protocol of Cholesterol LabAssay kit (Wako).

Supplemental References

1. Cretenet G, Le Clech M, Gachon F. Circadian Clock-Coordinated 12 Hr Period Rhythmic Activation of the IRE1α Pathway Controls Lipid Metabolism in Mouse Liver. Cell Metab 2010;11:47-57.
2. Preitner N, Damiola F, Luis-Lopez-Molina, Zakany J, Duboule D, Albrecht U, Schibler U. The Orphan Nuclear Receptor REV-ERBα Controls Circadian Transcription within the Positive Limb of the Mammalian Circadian Oscillator. Cell 2002;110:251-260.
3. Brown SA, Ripperger J, Kadener S, Fleury-Olela F, Vilbois F, Rosbash M, Schibler U. PERIOD1-Associated Proteins Modulate the Negative Limb of the Mammalian Circadian Oscillator. Science 2005;308:693-696.
4. Fonjallaz P, Ossipow V, Wanner G, Schibler U. The two PAR leucine zipper proteins, TEF and DBP, display similar circadian and tissue-specific expression, but have different target promoter preferences. EMBO J 1996;15:351-362.
5. Van Gelder RN, von Zastrow ME, Yool A, Dement WC, Barchas JD, Eberwine JH. Amplified RNA synthesized from limited quantities of heterogeneous cDNA. Proc Natl Acad Sci U S A 1990;87:1663-1667.
